# *Coxiella burnetii* infections from animals and ticks in South Africa: a systematic review

**DOI:** 10.1007/s11259-023-10204-z

**Published:** 2023-08-29

**Authors:** Letlhogonolo J. Diseko, Ana M. Tsotetsi-Khambule, ThankGod E. Onyiche, Tsepo Ramatla, Oriel Thekisoe, Nomakorinte Gcebe

**Affiliations:** 1 Unit for Environmental Sciences and Management, North-West University, Potchefstroom, 2520 South Africa; 2 Department of Life and Consumer Sciences, College of Agriculture and Environmental Sciences, University of South Africa, Florida, 1709 South Africa; 3 Department of Veterinary Parasitology and Entomology, University of Maiduguri, P.M.B. 1069, Maiduguri, Nigeria; 4 Agricultural Research Council–Bacteriology and Zoonotic Diseases Diagnostic Laboratory, Onderstepoort Veterinary Research, Onderstepoort, Pretoria, 0110 South Africa

**Keywords:** *Coxiella burnetii*, Domestic animals, Wildlife, Zoonotic pathogen

## Abstract

*Coxiella burnetii* is a zoonotic intracellular bacterium that is widely distributed and affects domestic animals, wildlife, humans and non-mammalian species. This systematic review was aimed at synthesizing research findings on *C. burnetii* in both domestic and wild animals of South Africa. The systematic review protocol was registered with Open Society Foundations of systematic reviews (10.17605/OSF.IO/8WS). PRISMA guidelines were followed to collect and evaluate relevant scientific articles published on *C. burnetii* infecting domestic and wild animals in South Africa. Published articles were sourced from five electronic databases, namely, Google Scholar, PubMed and ScienceDirect, EBSCO and Scopus. Results showed 11 eligible studies involving four domestic animals, three wild animals and one ectoparasite species from seven provinces across South Africa. The occurrence of *C. burnetii* infection was high in *Ceratotherium simum* (white rhinoceros) (53.9%), medium in sheep (29.0%) and low in pigs (0.9%). Limpopo province (26%) had the most recorded infections followed by KwaZulu-Natal (19%) and Free State (3%) had the least reported occurrence of *C. burnetii.* The current study discovered that there is scarcity of published research on prevalence and distribution of *C. burnetii* infecting domestic and wild animals in South Africa, and this is of concern as this bacterium is an important zoonotic pathogen of “One Health” importance.

**Supplementary Information:**

The online version contains supplementary material available at 10.1007/s11259-023-10204-z.

## Introduction

*Coxiella burnetii* is a zoonotic aerobic gram-negative intracellular bacterium capable of using numerous hosts of invertebrate and vertebrate hosts as vectors and reservoirs of infection respectively (Frean and Blumberg 2007). This pathogenic bacterium is widely distributed and can infect large number of hosts such as domestic animals (livestock and pets), wildlife and non-mammalian species (birds, reptiles and ticks) (Norlander 2000; Cutler et al. 2007). It can survive in two transmission cycles that are independent, but occasionally overlap (Norlander 2000). The first transmission cycle involves wildlife and their ectoparasites whereas the second involves domestic animals (Norlander 2000). Ruminants such as cattle, sheep and goats are commonly linked to human infections (Cutler et al. 2007). Ticks play a major role as carriers of *C. burnetii* infection in animals (Norlander 2000), but their chances of transmission from one animal to another or to humans are presumed to be lower (Körner et al. 2021). Adult animals infected with *C. burnetii* often remain systemically healthy but can have abortion and weak offspring (Roest et al. 2012). Infections of *C. burnetii* in humans may result from direct and indirect path (Roest et al. 2012). Direct path involves transmission of infection from animals to humans when the bacteria is shed in birth products (Roest et al. 2012; Eldin et al. 2017) and consumption of unpasteurized milk (Njeru et al. 2016). The most common indirect infection route is via inhalation of *C. burnetii* from the air which can be transported by wind over long distances (Boden et al. 2014). *Coxiella burnetii* causes Q fever in humans and symptoms include flu-like illness, pneumonia, headache, and reproduction problems or the infection can also be asymptomatic (Raoult et al. 2000).

The first clinical case report of Q fever in South Africa was from a human in 1950, where a boy child was clinically diagnosed with atypical pneumonia and was seropositive for *C. burnetii*, but the organism was not isolated (Gear et al. 1950). In 1976, a large investigative study on cattle and sheep was conducted in various parts of South Africa to determine which bacterial and viral agents were responsible for their perinatal loss (Schutte et al. 1976). Results showed that *C. burnetii* was present in the smears of collected placental tissues of cattle and sheep of different herds (Schutte et al. 1976). Since that time, there has not been a publication about the detection of Q fever and its causal pathogen (*C. burnetii*) using molecular techniques in South Africa until 2015 and 2019 where *C. burnetii* was detected from ticks collected from domestic animals (Mtshali et al. 2015; Guo et al. 2019). Mangena et al. (2021) reported the first genetic diversity of *C. burnetii* strains that circulate in domestic animals in South Africa. This systematic review seeks to provide updated scientific evidence on cases of *C. burnetii* in South Africa in relation to all involved hosts. This study further gives basic understanding on how this zoonotic pathogen is spread across domestic and wild animals of South Africa over a period of time and the risk factors involved.

## Materials and methods

### Review protocol

The systematic review protocol was registered with Open Society Foundations of systematic reviews (DOI: 10.17605/OSF.IO/8WS). The study was conducted following the principles of the Preferred Reporting Items for Systematic Review and Meta-Analysis (PRISMA) guidelines (Page et al. 2021). The purpose of this review was to identify, collect and assess all relevant scientific articles published in English with empirical data as a form of evidence for the presence of or distributional pattern of *C. burnetii* infections among domestic and wild animals found across all the nine provinces of South Africa. The review also aimed at compiling scientific findings which will serve as a point of reference to address questions of existing evidence on the presence of *C. burnetii* infections in animals found across South Africa, types of host species which are commonly affected by *C. burnetii* and provinces that are likely to have high or low rate of *C. burnetii* infection. Below are steps which were followed to identify published article/study records that investigated or researched cases of Q fever or occurrences of *C. burnetii* in domestic and wild animals across South Africa.

### Literature search

Literature search was conducted from 1st October 2021 to 30th November 2021. All the relevant titles and abstract with full texts were downloaded through library resources and online platforms, and no additional information nor unpublished articles were retrieved. The literature search used five search engines, namely Google Scholar, PubMed, ScienceDirect, EBSCO and Scopus using relevant key and Boolean operators (AND, OR) as follows; Prevalence of Q fever in South Africa, OR Prevalence of *Coxiella burnetii* in South Africa, Prevalence of Q fever in domestic AND wild animals of South Africa, Prevalence of *Coxiella burnetii* in domestic AND wild animals. Keywords that were targeted were *Coxiella burnetii*, Q fever, livestock, animals, sheep, goats, cattle, pigs, wild animals, prevalence AND South Africa. Identified title and abstracts were further used to select relevant full text articles for further investigation which was determined by a predefined criterion.

### Eligibility criteria

#### Inclusion criteria

In this systematic review, a published article was included if:

- its focus was on *C. burnetii* infection on domestic and wild animals, as well as ticks,
- specifically showed the prevalence of *C. burnetii* infecting domestic and wild animals, as well as ticks across provinces in South Africa,
- its diagnostics were made using either serological or molecular technique such as conventional PCR, real-time PCR, etc.,
- the publication was written in English language.

#### Exclusion criteria

A record was excluded in this systematic review using the following guide:

- not focused on *C. burnetii* infections on domestic and wild animals, as well as ticks in South Africa,
- focused on *C. burnetii* infections on humans,
- focused on the prevalence of *C. burnetii* infecting domestic and wild animals of other countries than South Africa,
- not written in English and not published in a peer-review journal,
- had no clear methods and materials, and sample records,
- a dissertation, thesis or review article.

### Assessment and selection criteria

The first phase was to use title and abstract to identify records from an online data-base and to remove duplicates and irrelevant records. All the retrieved relevant articles according to titles were further screened based on the abstracts to identify relevant articles. If a record occurred where a study was conducted on animals and humans, preference was given to animals. In records that included animals, but the study was unpublished dissertation, thesis or published short communication without a clear methodology, the records were excluded.

### Estimation of prevalence

The prevalence of *C. burnetii* in animals was calculated based on numbers of given individual species and not from a taxonomic reference. The retrieved data regarding prevalence of host species was further analyzed with doughnut graph and bubble map on Microsoft Excel to illustrate the estimates of *C. burnetii* prevalence across the nine provinces of South Africa.

### Risk of bias analysis

Systematic bias or error is any process that infers at any stage of the study by causing results to differ systematically from the real values (Linares-Espinós et al. 2018). Thus, poor quality of the study can compromise and invalidate the outcome of the systematic review (Linares-Espinós et al. 2018). There are several tools which can be used to examine the quality of the study which include study design, publication bias, imprecision, selection bias, allocation bias, detection bias, attribution bias and reporting bias (Nezaratizade et al. 2022). Eleven articles were included and analyzed for quality to avoid the risk of bias in the study. Articles were analyzed based on a self-made questionnaire and quality eligibility criteria according to the JBI critical appraisal checklist (JBI Evidence Synthesis Manual) and guidelines in the Grade handbook and the Cochrane handbook for systematic reviews of interventions (Higgins et al. 2022). The risk assessment was conducted by evaluating the following variables; location (province), publication year, type of samples, host species and methods. The quality of relevant studies was evaluated by a system of scoring through a modified checklist that was based on the following questions: (i) was the animal frame appropriate to address the target population? (ii) was the animal sample randomly selected from the population? (iii) were the animals randomly selected and randomly allocated? (iv) was the title and abstract of the study relevant? (v) were the methods and data analysis of the study clear and sufficiently covered the identified animals? (vi) does the study give detailed analysis about the statistical approach and dataset analysis? (vii) were the study participants sampled in an appropriate way? (viii) was the sample size adequate? (ix) was the interpretation of the results and discussion taking account of the study aims/objectives? Each paper was assessed based on these nine questions and scored at 9/9 × 100% (Supplementary table S1). The score that was above 70% was considered to be of high-quality paper, between 69 and 50% average quality paper and below 49% low quality paper (16). Thus, no paper was omitted from this review paper based on the scores, because any score that was obtained reflected the quality of the study paper on *C. burnetii* infections in domestic and wild animals, as well as ticks of South Africa, but not the quality of this review.

## Results

**Fig. 1:**
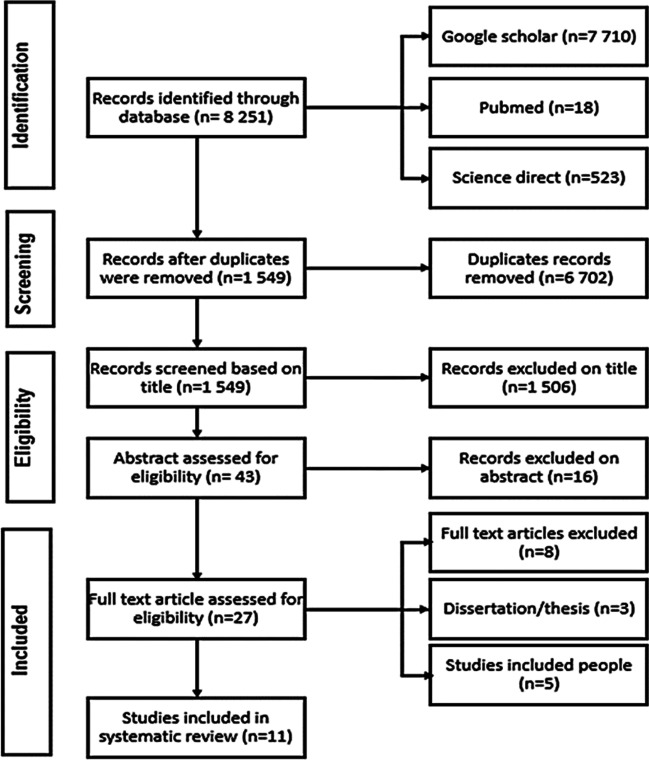
PRISMA flow chat illustrating selection criteria of articles used in this review paper

From an online database search, a total of 8251 publications was obtained for this review paper. Of those, 6702 publications were removed. The number of records that were deemed as eligible based on their title was 1549 and 1506 were excluded based on further analysis of their title. Further assessment on the remaining 43 records was performed based on their abstracts, resulting in the removal of 16 more. The remaining 27 study records were then assessed based on evaluation of their full text. A further eight studies, from three dissertations/thesis and five human studies, were removed. Thus, the final number of study records/articles that were included in this systematic review totals eleven (*n* = 11) (Fig. 1). The eleven eligible studies were distributed across seven South African provinces, namely; North West, Gauteng, Limpopo, Free State, Mpumalanga, KwaZulu-Natal and Eastern Cape (Table 1). Animal samples that were analyzed within the studies used here were domestic, such as cattle, sheep, cats and pigs and wildlife included wild dogs and white rhinos as well as invertebrates such as ticks (Table 1).

**Table 1:** List all the key studies selected for systematic review of *Coxiella burnetii* in animals in South Africa

Provinces	Year of publication	Type of Samples	Host/Vector (s) screened	Diagnostic Methods	No. of PCR positive	Molecular prevalence (%)	No. of seropositive	Seroprevalence (%)	References
Gauteng, North West, Limpopo & Mpumalanga	1976	Serum	Cattle (*n* = 80) Sheep (*n* = 31)	Complement Fixation Test (CFT) Micro-agglutination test (CR)	−*	–	91	81.9%	Schutte et al. 1976
Gauteng	1987	Serum	Cattle (*n* = 8900)	CFT	–	–	962	7.7%	Gummow et al. 1987
Limpopo	1995	Serum	Wild dogs (*n* = 16)	ELISA	–	–	16	100%	Van Heerden et al. 1995
Not specific	1997	Serum	Cats (*n* = 171)	Immunofluorescent Antibody (IFA)	–	–	171	10.0%	Matthewman et al. 1997
Not specific	2015	Tissue	Ticks (*n* = 590)	PCR	41	6.9%	–	–	Mtshali et al. 2015
Limpopo Western Cape	2015	Tissue	Ticks (*n* = 205)	PCR	0	0%	–	–	Halajian et al. 2016
Free State, KwaZulu-Natal, North West, Mpumalanga	2017	Tissue	Ticks (*n* = 149)	PCR	57	38.3%	–	–	Mtshali et al. 2017
Free State	2019	Tissue	Ticks (*n* = 61)	PCR	1	1.6%	–	–	Guo et al. 2019
Eastern Cape	2019	Tissue	Ticks (*n* = 42)	PCR	11	26.2%	–	–	Guo et al. 2019
KwaZulu-Natal	2019	Tissue	Ticks (*n* = 27)	PCR	0	0%	–	–	Guo et al. 2019
Mpumalanga	2020	Serum	Cattle (*n* = 184)	CFT	–	–	70	38.0%	Adesiyun et al. 2020
Limpopo	2021	Serum	White rhinos (*n* = 89)	IFA	–	–	48	53.93%	Donnelly et al. 2021
Gauteng and Free State	2021	Serum & Tissue	Cattle (*n* = 331) Sheep (*n* = 69) Pigs (*n* = 107)	PCR and ELISA	63	12.7%	35	6.9%	Mangena et al. 2021

*Not done

**Fig. 2:**
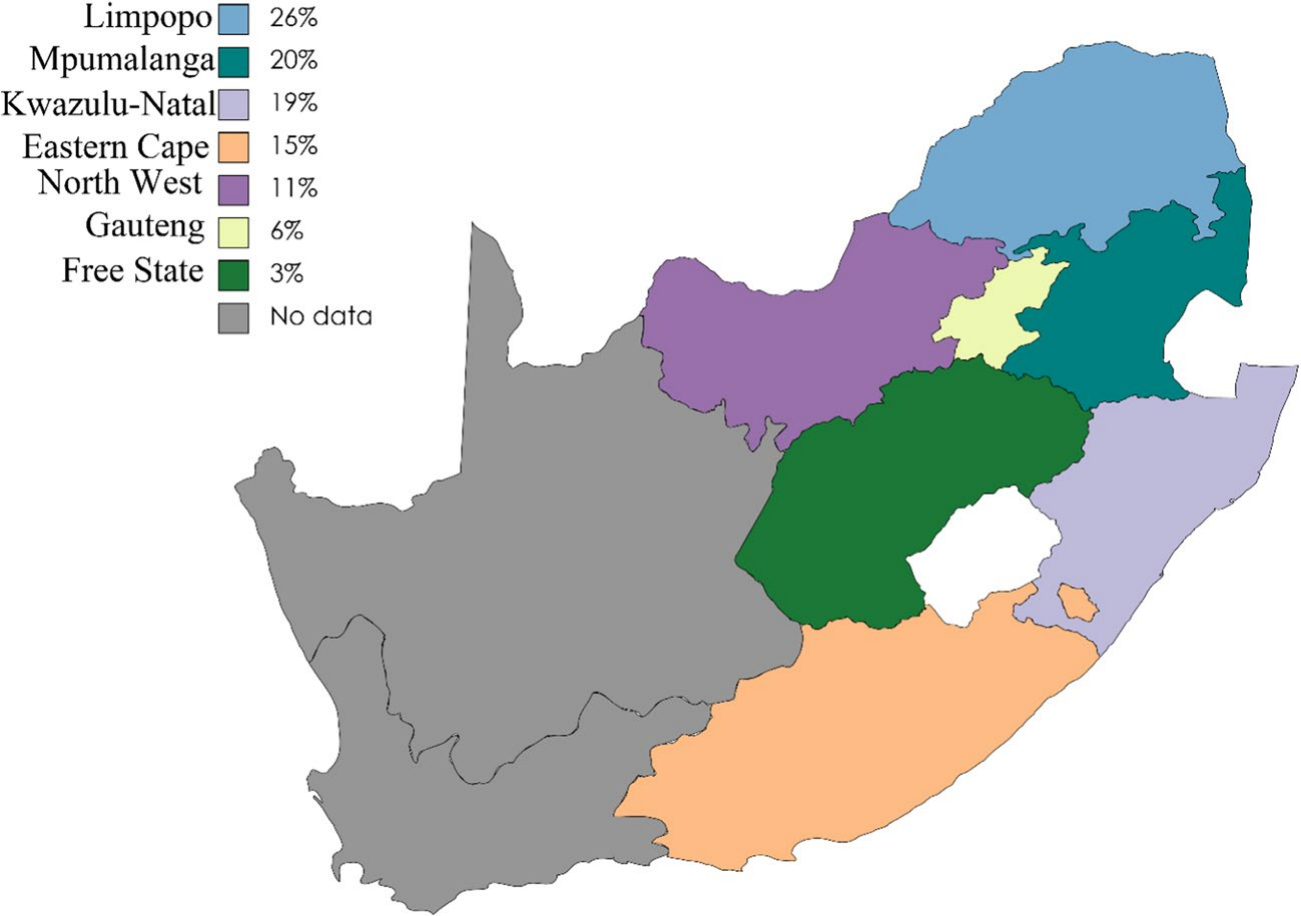
Prevalence of *Coxiella burnetii* in South African provinces. [https://www.mapchart.net/world.html (accessed on 04 May 2023)]

In terms of year of publication, the study records used in this review are from 1976 to 2021 (Table 1). Based on the diagnostic approach employed, seven studies used serological tests, three studies used molecular techniques and only one study used molecular technique to detect *C. burnetii* in animals across provinces of South Africa. Overall occurrences of *C. burnetii* exposure was high in Limpopo (26%), followed by Mpumalanga (20%), KwaZulu-Natal (19%), North West (13%), Gauteng (6%) and Free State (3%) (Fig. 2). However, an overall illustration for *C. burnetii* across the provinces based on the detection of the pathogen was not included, because it narrows down to only four studies on PCR technique, where one study did not specify its location/province sampling (Mtshali et al. 2015) and the other study used pooled DNA samples of sheep organs form the Free State province (Mangena et al. 2021) (Tables 1 and 2). In terms of host species wild dogs (100%) and white rhinoceros (53.9%) had high seroprevalence *C. burnetii* (Table 2), followed by sheep, cats, cattle and pigs with 29.0%, 9.9%, 8.9% and 0.9% respectively. There were no published studies on *C. burnetii* infections on domestic animals, wildlife and ticks from the Western Cape and Northern Cape provinces.

**Table 2:** Overall occurrences of *C. burnetii* in South Africa according to host species

Host types	Serology detection			Molecular detection		
	Sample size	Positive	Seroprevalence (%)	Sample size	Positive	Prevalence (%)
Cattle	9495	858	8.9%	63	6	9.5%
Sheep	98	29	29.0%	63	2	3.2%
Pigs	107	1	0.9%	–	–	–
White rhinos	89	49	53.9%	–	–	–
Wild dogs	16	16	100%	–	–	–
Cats	171	17	9.9%	–	–	–
Ticks	130	12	9.2%	1074	110	10.24%
Total	10,106	982	9.7%	1200	118	9.83%

## Discussion

*Coxiella burnetii* can infect both animals and humans (Małaczewska et al. 2018), and its high infectivity, environmental stability and aerosol transmission present it as a potential biological threat (Long et al. 2019). Thus, the data reviewed in this study revealed that *C. burnetii* infections have been detected from various animal hosts and ticks from different sites throughout South Africa. We observed that only one study investigated *C. burnetii* infection in both domestic and wild animals which was conducted in the 1970s by Schutte et al. (1976). In the 1980s, Gummow et al. (1987) investigated Q fever on cattle and after that there were no published studies in the periods of 1990–1999 and 2000–2014. However, recently [2015–2021], there has been an increase in *C. burnetii* prevalence studies conducted on animals and ticks in South Africa, due to the use of more sensitive and specific tests, such as ELISA for detection of antibodies and PCR detection of the pathogen DNA. Studies included in this review have mainly used serological assays including CFT, ELISA, IFAT and microagglutination test to determine occurrence of *C. burnetii* infections. The most commonly used indirect method worldwide is IFA due to its accuracy, high specificity and sensitivity (Porter et al. 2011). The CFT less specific and less sensitive compared to ELISA, but is recommended by OIE in veterinary medicine as a serological diagnostic (Porter et al. 2011). The advantage of serological assays is that they can be used in large scale epidemiological surveys to indicate exposure of animals or humans to infection, but cannot be applied for detection of pathogen from the arthropod vector. Other studies included in this systematic review have used PCR for detection of *C. burnetii* infections, which is a direct diagnostic method which detects presence of bacterial DNA and has advantage of been applied for detection from arthropod vectors as well. Our study observed that in terms of diagnostic methods used by primary studies a low detection of *C. burnetii* was from PCR (5.65%) and CFT (7.88%). The highest detection of *C. burnetii* infections was from IFA with 25% and micro-agglutination 82.18%. Studies that used ELISA methods had a medium detection rate of 17.11%. Such variation is mainly because indirect methods (serological assays) detect antibodies which can be present during and after clearance of the pathogen, hence they display much higher infections rates. A direct method such as PCR accurately indicates presence of infection, hence it tends to give lower infection rates.

There are no existing published studies on *C. burnetii* infections on domestic and wild animals from Northern Cape and Western Cape provinces. In terms of hosts, high seroprevalence (53.9%) of *C. burnetii* infection were recorded from white rhinoceros private reserves in Cullinan, Lichtenburg, Lydenburg, Middelburg, Polokwane, Thabazimbi, and Vaalwater. In other parts of the world *C. burnetii* has been detected from a placental tissue of white rhinoceroses in the United States (Bercier et al. 2021). Rhinos are listed as endangered species under the conservation status of red list species (IUCN 2006) and infections of *C. burnetii* can lead to clinical signs such as reproductive failure in wildlife (Clemente et al. 2009), thus a combination of such factors might cause the rhinoceros species to struggle to rehabilitate if not well managed. Donnelly et al. (2021) explained that the source of infection to rhinos is unknown, but ticks were most likely the suspects, serving as vector for transmission of the infection.

About 40 species of ticks have been found to harbor *C. burnetii* infection (Spitalská and Kocianová, 2003) and *Coxiella*-like endosymbionts in some tick species (Klyachko et al. 2007). Our study observed low prevalence of *C. burnetii* infection in ticks (9.2%) which was higher than that reported from Kenya (2.5%) and Ethiopia (6.4%) (Mediannikov et al. 2010), but lower than that of Nigeria (14.0%) (Reye et al. 2012). Thus, risk factors that may arise from *C. burnetii* in ticks as observed by our studies include increased chances of contamination of the environment as a result of possible excretion of the bacterium through tick faeces, saliva and coxal fluids (Maurin and Raoult 1999; Menadi et al. 2020). Furthermore, infected ticks might transmit this bacterium to other animals during blood feeding (Mediannikov et al. 2010). However, in South Africa studies on *C. burnetii* occurrence in tick populations relative to animals, vegetation and environment from dip tanks, farms, abattoirs, national parks and private game reserves remains poorly covered which further limits our understating of the transmission dynamics and distribution of this zoonotic pathogen in wild and domestic animals of South Africa.

Our study observed a low prevalence of *C. burnetii* infection in cattle (8.9%) which is slightly similar to 7.0%, 10.5% and 11.3% registered from cattle in Thailand, Kenya and Algeria respectively (Menadi et al. 2020). Our observation suggests that cattle in South Africa are exposed to *C. burnetii* infections, and we estimate that suspected cases in cattle might be related to reproductive disorder or abortions and thus warrants further investigation to determine the possible incrimination of *C. burnetii*. Recently, De Boni et al. (2022) reported the possible exposure of abattoir workers to *C. burnetii* in the Free State and Northern Cape provinces which might be due to inhalation or close contact with aerosolized *C. burnetii*. From the published reports on *C. burnetii* infections on cattle from South Africa, there are only four published reports or articles which included cattle and from them only one study used both ELISA and PCR, whereas the rest of the three studies used serology (Table 1). None of the four papers included cattle samples from Eastern Cape and KwaZulu-Natal provinces, which according to Department of Agriculture, Land Reform and Rural Development (DALRRD) annual report for 2019–2020 have been listed as the highest producers of cattle in South Africa (www.dalrrd.gov.za). The highest record of *C. burnetii* seroprevalence from cattle in South Africa was during 1976 which was 81.2% (Schutte et al. 1976) and in 2020 Adesiyun et al. (2020) recorded the second highest (38%) ever infection of *C. burnetii* on cattle in South Africa. Though both studies, Schutte et al. (1976) and Adesiyun et al. (2020) used a serological assay (CFT) and have a large 44-year period between them, the huge drop difference in seroprevalence on cattle might be due to decreased exposure. Recently, Mangena et al. (2021) recorded *C. burnetii* infection rate of 9.4% using ELISA, thus such disparities of *C. burnetii* infections in cattle might reflect the use of different testing tools with different sensitivity and specificity.

A survey conducted by Gummow et al. (1987) in Gauteng, Mpumalanga and Limpopo provinces, which were then called Transvaal, recorded a seroprevalence of 7.7% for *C. burnetii* in cattle and they also suspected that this region could have more exposure of this zoonotic pathogen than what they recorded. Taking all of this into consideration regarding cattle exposure to *C. burnetii* and the high population of cattle in the Eastern Cape, KwaZulu-Natal and Free State provinces, there in a need to implement more awareness program and improved polices, such as mandatory surgical musk on people working with livestock/meat (e.g. feedlot or abattoirs) and keeping high standards of hygiene in such facilities in order to protect them from possible aerosol infections (De Boni et al. 2022).

This study also observed a low prevalence of *C. burnetii* in cats (9.9%) (Matthewman et al. 1997) and remains to be the only published study in South Africa. There are no other studies that have been conducted to screen for this zoonotic bacterium from cat tissue, serum or DNA samples for over the past 25 years, with the exception to Mtshali et al. (2017) who detected *C. burnetii* on ticks collected from cats. When compared to other studies from other countries, our study observed that the prevalence of *C. burnetii* infections in cats [9.9%] (Matthewman et al. 1997) from South Africa is higher than reported prevalence in Turkey (4.9%) (Kiliç et al. 2008) and Korea (8.6%) (Komiya et al. 2003), but lower than that from Japan (Komiya et al. 2003) and Australia (Ma et al. 2020) with 14.2% and 13.1% respectively. Companion animals such as cats and dogs have shown evidence of being sources of infections to humans (Htwe et al. 1992). There has been a recorded seroprevalence of 0.9% in pigs in South Africa by a study conducted by Mangena et al. (2021). However, there is no scientific evidence that confirms zoonotic cases of *C. burnetii* between pigs and humans, because pigs are either rarely exposed to *C. burnetii* or their epidemiological data is limited (Seo et al. 2016). Though prevalence of *C. burnetii* in pigs is commonly low, our study observed that *C. burnetii* infection in pigs (0.9%) from South Africa is lower than prevalence reported in South Korea (6.8%) (Seo et al. 2016) and China (3.0%) (S. El-Mahallawy et al. 2016). In South Africa, pigs represent the smallest industry in the overall agricultural sector and its highest production areas are in Limpopo and North West provinces, but its production has continued to increase in past years because it is the most affordable and consumed meat (www.dalrrd.gov.za). Sheep, goats and cattle are regarded as possible reservoirs of *C. burnetii* and source of infections to humans (Maurin and Raoult 1999). The single largest transmission event of *C. burnetii* from animals to humans occurred in dairy goats in the Nertherlands (Roest et al. 2011). In Germany, a flock of sheep was linked to source of infection for Q fever that occurred among rural town residents (Lyytikäinen et al. 1998). In Italy, prevalence of *C. burnetii* infection in humans was connected to a flock of sheep as a source of infections (Manfredi Selvaggi et al. 1996). A study by Mtshali et al. (2015) detected *C. burnetii* infection (32%) from ticks collected from sheep in South Africa using PCR. Two serological studies reported prevalence of *C. burnetii* from sheep sera in South Africa. The time overlap between the two studies is about 45 years, with a wide margin of decrease of prevalence from 83.8% (Schutte et al. 1976) to 4.3% (Mangena et al. 2021).

We hypothesized that this huge decline in *C. burnetii* prevalence on sheep could have been influenced by different testing tools of serology versus molecular technique which have different levels of sensitivity and specificity. However, it should be noted that the two studies being compared, Schutte et al. (1976) and Mangena et al. (2021), are quite dissimilar as the former was based on testing placentas from farms with abortion. Despite scarce and low reports of *C. burnetii* infection in small stock in South Africa, there are numerous studies which have reported prevalence of *C. burnetii* infections from sheep in Guinea (2.3%) (Troupin et al. 2022), Egypt (8.9%) (Klemmer et al. 2018), Gambia (18.5%) (Klaasen et al. 2014), Turkey (29.0%) (Parin and Kaya 2015), and Spain (31.7%) (Rodríguez et al. 2010).

This study also noted that wild animals such as wild dogs have been reported to have *C. burnetii* infections during 1976, but the sample size was low (<20) making it difficult to make any comparison with other studies. This low number of *C. burnetii* infections in wild animals may be linked to difficulties in sampling and reporting free-ranging wild animals (Mörner et al. 2002) or low-level clinical morbidity resulting in a lack of studies (Arricau-Bouvery and Rodolakis 2005). Lastly, our study has observed a variety of pattern changes in terms of the prevalence of *C. burnetii* infection with regard to provinces involved. For example, high prevalence was documented from studies in provinces such as Limpopo, Mpumalanga and KwaZulu-Natal. Moderate occurrences were from Eastern Cape and North West, and low occurrences from Free State and Gauteng. However, such occurrences might reflect a geographical distribution of this zoonotic pathogen as a result of specific outbreaks in different provinces or specific findings from different research groups working in different provinces over a period of 45 years.

## Conclusion

This study has demonstrated that there are few studies with interest on *C. burnetii* infections on wild and domestic animals of South Africa. To date South Africa has about 431 registered abattoirs [Find the DALRRD list of abattoirs (2021, November) on its website, www.dalrrd.gov.za], 19 South African National Parks, over 40 private game reserves and countless livestock farms across nine provinces, but has only eleven published articles which investigated *C. burnetii* infections on wild and domestic animals, as well as ticks in a period of 5 decades. This points out the need to conduct more epidemiological studies for *C. burnetii* infections throughout the country to establish the impact and risk factors it might have across various animals of South Africa. There is also a need to increase resources and human capital in order to have an integrated surveillance of *C. burnetii* on national parks and private nature reserves, which play a role in tourism attraction, and abattoirs and animal farms, which are crucial for food production and safety in South Africa.

### Supplementary Information

ESM 1 (DOCX 18 kb)

## Data Availability

Research data is available upon receipt of a reasonable request.
