# Supplementary material for: Coxiella burnetii infections from animals and ticks in South Africa: a systematic review
Source: Vet Res Commun. 2023 Aug 29;48(1):19–28. doi: 10.1007/s11259-023-10204-z (PMC10811037; doi:10.1007/s11259-023-10204-z)
Supplement: Supplementary file 1 — (DOCX 18 kb) [file 11259_2023_10204_MOESM1_ESM.docx]

***Coxiella burnetii* infections from animals and ticks in South Africa: A systematic review**

Letlhogonolo J. Diseko^1^, Ana M. Tsotesti-Khambule^2^, ThankGod E. Onyiche^3^, Tsepo Ramatla^1*^, Oriel Thekisoe^1^ and Nomakorinte Gcebe^4^

^1^Unit for Environmental Sciences and Management, North-West University, Potchefstroom, 2520, South Africa.

^2^Department of Life and Consumer Sciences, College of Agriculture and Environmental Sciences, University of South Africa, Florida, 1709, South Africa.

^3^Department of Veterinary Parasitology and Entomology, University of Maiduguri, P.M.B. 1069, Maiduguri, Nigeria.

^4^Agricultural Research Council–Bacteriology and Zoonotic Diseases Diagnostic Laboratory, Onderstepoort Veterinary Research, Onderstepoort, Pretoria 0110, South Africa.

***Corresponding author**: Tsepo Ramatla, Email: [a21205450@gmail.com](mailto:a21205450@gmail.com)

**Supplementary Table S1 The Joanna Briggs Institute (JBI) critical appraisal checklist for studies reporting prevalence data on *Coxiella burnetii.***

|  | References | 1 | 2 | 3 | 4 | 5 | 6 | 7 | 8 | 9 | Total | score |
| --- | --- | --- | --- | --- | --- | --- | --- | --- | --- | --- | --- | --- |
| 1 | Schutte *et al*., 1976 | Y | Y | Y | Y | Y | N | Y | Y | Y | 8/9 | 88.88% |
| 2 | Gummow *et al*., 1987 | Y | Y | Y | Y | Y | N | Y | Y | Y | 8/9 | 88.99 |
| 3 | van Heerden *et al*., 1995 | N | N | N | Y | N | N | Y | N | N | 1/9 | 11.11% |
| 4 | Matthewman *et al*., 1996 | Y | Y | Y | N | N | N | Y | Y | N | 3/9 | 33.33 |
| 5 | Halajian *et al*., 2015 | Y | Y | N | Y | Y | Y | Y | Y | Y t | 8/9 | 88.88% |
| 6 | Mtshali *et al*., 2015 | Y | Y | Y | Y | Y | Y | Y | Y | Y | 9/9 | 100% |
| 7 | Mtshali *et al*., 2017 | Y | Y | Y | Y | Y | Y | Y | Y | Y | 9/9 | 100% |
| 8 | Guo *et al.*, 2019 | Y | Y | Y | Y | Y | Y | Y | Y | Y | 9/9 | 100% |
| 9 | Donnelly *et al*., 2020 | Y | Y | Y | Y | Y | Y | Y | Y | Y | 9/9 | 100% |
| 10 | Adesiyun *et al*., 2020 | Y | Y | Y | Y | Y | Y | Y | Y | Y | 9/9 | 100% |
| 11 | Mangena *et al*., 2021 | Y | Y | Y | Y | Y | Y | Y | Y | Y | 9/9 | 100% |
|  |  |  |  |  |  |  |  |  |  |  | 82/99 | 82.82% |

**The checklist questions to determine the risk of bias for the included studies**:

1. Was the animal frame appropriate to address the target population?

2. Was the animal size randomly selected from the population?

3. Were the animals randomly selected and randomly allocated?

4. Was the title and abstract of the study described in detail?

5. Were the methods and data analysis of the study clear and sufficiently covered the identified animals?

6. Does the study give full detail analysis about the statistical approach and dataset analysis?

7. Were the study participant sampled animals in an appropriate way?

8. Was the sample size adequate?

9. Was the interpretation of the results and discussion relating to the study aims/objectives?
